# L’association entre les gènes HLA II et la susceptibilité à l’asthme chez une population constantinoise sélectionnée

**DOI:** 10.11604/pamj.2020.35.48.13340

**Published:** 2020-02-18

**Authors:** Dahbia Ines Dahmani, Nacima Chila, Fouzia Abdelouahab, Houda Bouyoucef, Mohamed Bougrida, Laila Rouabah, Fayssal Nedjar

**Affiliations:** 1 Unité HLA Service de Physiologie et Exploration Fonctionnelle, CHU Benbadis, Constantine, Algérie; 2 Laboratoire de Recherche en Maladies Métaboliques, Faculté de Médecine, Université de Constantine 3, Algérie; 3 Laboratoire de Biologie Bellulaire et Moléculaire, Faculté des Sciences de la Nature et de la Vie, Université des Frères Mentouri, Constantine 1, Algérie; 4 Service de Physiologie, CHU de Blida, Blida, Algérie

**Keywords:** HLA, HLA classe II, asthme, HLA key, HLA classe II, asthma

## Abstract

**Introduction:**

l’asthme est une maladie complexe causé par l’interaction de plusieurs gènes dont certains ont un effet protecteur et d’autres contribuent à l’apparition de la maladie. Cet article s’intéresse à une partie du bras court du chromosome 6 où siègent les gènes codant pour le complexe majeur d’histocompatibilité dénommé HLA qui joue un rôle important dans la pathogénèse de l’asthme.

**Méthodes:**

notre étude inclut 61 personnes dont 30 personnes non asthmatiques non allergiques et 31 patients atteints d’asthme allergique depuis plus de 2 ans. Le statut atopique de l’asthme a été confirmé par les tests cutanés et le typage HLA classe II a été réalisé suivant le principe de la technique sérologique de séparation par Fluorobeads B.

**Résultats:**

l’analyse statistique révèle que les gènes HLA-DR11 et DQ2 sont significativement plus fréquents chez les asthmatiques par rapport aux témoins, les résultats sont comme suit: le HLA-DR11 avec une p=0,05 et le HLA-DQ2 avec une p=0.002, cela nous a conduit à dire qu’il s’agit peut-être de marqueurs HLA en association avec l’asthme. En revanche le marqueur HLA-DQ6 était nettement plus important chez la population témoin que chez les sujets asthmatiques avec p=0.003.

**Conclusion:**

cela nous a conduit à dire que les marqueurs HLA-DR11 et DQ2 pourraient être des gènes potentiels de susceptibilité à l’asthme, alors que HLA-DQ6 pourrait avoir un effet protecteur.

## Abstract (en)

**Introduction:**

asthma is a complex disease caused by the interaction of several genes. Some of them have a protective effect and some others contribute to disease onset. This study focuses on a region of the short arm of chromosome 6, where the genes encoding the major histocompatibility complex called HLA are located. This short arm plays an important role in the pathogenesis of asthma.

**Methods:**

our study included 61 subjects, 30 non-asthmatic, non-allergic subjects and 31 patients with more than a 2-year history of allergic asthma. Atopic asthma was confirmed by skin tests and HLA-typing of class II which were performed following the principle of the serological technique of separation by FluoroBeads B.

**Results:**

statistical analysis showed that HLA - DR11 and DQ2 genes were significantly more frequent in the asthmatic group compared to the control. The results were as follows: HLA-DR11 with p=0.05 and HLA-DQ2 with p=0.002. Based on this results HLA markers could be associated with asthma. On the other hand HLA-DQ6 marker was substantially higher in the control group than in the asthmatic subjects with p=0.003.

**Conclusion:**

HLA DR11 and DQ2 markers could be potential markers of susceptibility to asthma, while HLA-DQ6 may have a protective effect.

## Introduction

L’asthme représente un problème majeur de santé publique. Il s’agit d’une maladie multifactorielle hétérogène résultant des effets et des interactions de nombreux facteurs génétiques et environnementaux [1]. C’est une pathologie qui mobilise plusieurs équipes de chercheurs à l’échelle mondiale, ce qui a pour effet d’augmenter les connaissances et de mieux comprendre son histoire naturelle. L’identification des déterminants génétiques s’effectue par des études d’épidémiologie génétique, comme des analyses de liaison ou des études d’association qui permettent de trouver des régions chromosomiques liées à l’asthme et des gènes associés à son développement. Parmi ces gènes, certains pourraient avoir un effet protecteur tandis que d’autres contribueraient à la pathogénèse de la maladie (gènes de susceptibilité) [2]. Notre présente étude est axée sur une partie et non des moindres du bras court du chromosome 6 où siègent les gènes codant pour le complexe majeur d’histocompatibilité humain dénommé HLA (Human Leukocyte Antigen) qui un est un système de groupes tissulaires qui joue un rôle important dans la reconnaissance du soi et du non soi, il est considéré comme une carte biologique permettant de différencier les individus entre eux. Il s’agit d’un système immuno-génétique très polymorphe, multigénique, multiallélique, d’expression codominante et aux fonctions immunitaires primordiales dans l’initiation et le contrôle de la réponse immune. De nombreux gènes extrêmement polymorphes ont été décrits au sein de ce système dont les gènes HLA de classe II (HLA-DR, DP et DQ) qui codent pour des molécules impliquées dans la présentation des antigènes étrangers aux récepteurs des lymphocytes T.

Ces études nous ont permis de mieux comprendre les mécanismes physiopathologiques de l’asthme avec l’implication de gènes codant des protéines décrites pour initier une réponse inflammatoire de profil Th2 (comme les gènes codant l’IL-33 et son récepteur IL1R1L) en réponse à des dommages tissulaires de l’épithélium bronchique. Dans ces mêmes études, une méta-analyse des résultats obtenus pour les IgE totales a mis en évidence cinq locus: FCER1A (1q23), IL13 (5q31), HLA-DRB1 (6p21), STAT6 (12q13) et IL4R/IL21R (16p12-p11). Seuls deux gènes étaient communs à l’asthme et aux IgE (IL13 et région HLA). L’objectif ultime visé par cette étude est d’évaluer l’association des différents typages HLA avec le statut allergique de l’asthme, et particulièrement hypersensibilité allergique qui pourrait être un facteur déclencheur et/ou aggravant de l’asthme. Les résultats attendus pourront être utiles dans l’avancement des travaux sur la génétique de l’asthme surtout au nord-africain et particulièrement en Algérie. Car cette maladie est très peu étudiée, et la dernière enquête TAHINA qui a été réalisé en 2007 par l’Institut national de la santé publique (INSP) qui révèle que les maladies respiratoires occupent la 2^e^ place des causes de morbidité (11,65%) et la première cause des motifs de consultation (25,33%). Et Ils l’ont classé au 3^e^ rang des maladies chroniques après l’HTA et le diabète en Algérie [3]. Même si beaucoup de travaux sont encore nécessaires en ce sens afin de comprendre les mécanismes physiopathologiques qui sous-tendent la maladie, l’objectif reste toujours le même c’est bien la précision de la définition de l’asthme et d’amélioration des conditions de vie des individus asthmatiques. La découverte de gènes de susceptibilité à l’asthme permettra certainement une meilleure prévention, un meilleur diagnostic et un meilleur traitement, lesquels seront éventuellement axés sur les prédispositions génétiques de chacun à développer de l’asthme.

## Méthodes

Notre étude immunogénétique transversale de type cas/témoins qui s’est étalée sur une période de 5 mois dans le cadre d’un mémoire de master. Cette étude a porté sur 61 personnes dont 30 personnes non asthmatiques non allergiques et 31 patients atteints d’asthme allergiques depuis plus de 2 ans orientés par leurs médecins traitants (pédiatres, pneumologues) au service de physiologie et exploration cardio-respiratoire (CHU-Constantine) pour contrôle d’asthme, ou ils étaient sollicités pour participer à l’étude (Les patients convaincus ont signé un consentement éclairé confidentiel qui comporte l’intérêt et l’objectif de l’étude). Afin d’assurer cette confidentialité, des codes ont été attribués pour chaque participant. Le typage HLA a été réalisé dans le laboratoire HLA du même CHU. Parmi les 31 asthmatiques on a 64,50% de femmes et 35,50% d’hommes, comportant toute catégorie d’âge à l’exception des enfants < 13 ans, avec une moyenne de 43,29±17,83 ans, le poids moyen de notre échantillon était de 75,90±14,98kg. Le groupe des témoins est composé de 30 sujets sains (non asthmatiques, non allergiques, non atopiques) avec 20% de femmes contre 80% d’hommes dont les moyennes étaient 40.36±18.50 ans pour l’âge et 78.20±17.81kg pour le poids. Après avoir signé le consentement éclairé pour être volontaire et participer à l’étude, les patients ont été examinés par le même médecin afin de confirmer le diagnostic d’asthme allergique.

**Exploration de la fonction respiratoire:** l’évaluation clinique du niveau de contrôle de l’asthme a été établie par le même médecin physiologiste selon les critères GINA 2008 qui consiste à évaluer l’état du patient sur les quatre dernières semaines qui sont: les symptômes diurnes et nocturnes, l’utilisation des bronchodilatateurs de courte durée d’action, la limitation de l’activité physique et l’exacerbation [4,5].

**Identification du statut allergique en utilisant les skins prick test et le dosage des IgE totales:** par la suite nos patients ont été tous orientés vers un médecin allergologue dont la sensibilisation était confirmée par la positivité d’au moins deux tests cutanés sur une batterie de 15 pneumallergènes (Laboratoires Stallergénes® Paris, France) en utilisant une procédure standardisée [6]. Pour confirmer cet état d’atopie, le taux des IgE a été fixé comme supérieur à 300 UI/ml. Les IgE ont été dosés par une technique immunoturbidimétrique (Biokit, SA, Barcelone, Espagne) en utilisant l’analyseur Architect CI 8200 (Abbott Laboratories, Abbott Park, IL, USA).

**Questionnaire et Typage HLA:** après la confirmation du statut allergique de l’asthme un prélèvement sanguin a été effectué par un infirmier au service de physiologie et exploration fonctionnelle et exploration à l’unité HLA. Les participants ont été menés à répondre un questionnaire sur leur santé respiratoire, actuelle et passée, il s’agit d’un questionnaire standardisé selon les critères de American Thoracic Society (ATS), modifié pour inclure la sévérité de l’asthme, l’allergie, l’atopie ainsi que l’histoire familiale de l’asthme et des autres maladies respiratoires (American Thoracic Society, 1987) [7]. Après le prélèvement 4ml de sang (prélèvement stérile) dans des tubes ACD qui sont fortement recommandés pour l’isolement avec les Fluorobeads. Ces dernières sont des billes immunomagnétiques d’un diamètre inférieur à 1 micron, elles disposent d’anticorps monoclonaux anti-CD19 attachés à leur surface. Les anticorps CD19 ne se lient qu’aux lymphocytes B. Ces billes permettent d’utiliser une méthode de séparation rapide des lymphocytes B du sang, en utilisant un séparateur magnétique pour le typage HLA Classe II en fluorescence, cette méthode ne nécessite aucune incubation à froid, agitation ou centrifugation. Le PBS/Citrate améliore la performance des billes. La première étape entamée dans notre étude immunogénétique est la reconstitution des plaques de Terasaki par l’addition de 1µl d’eau stérilisée et 5µl d’huile minérale dans chaque puits, ces plaques sont par la suite conservées à une température entre +2°C et +5°C pendant 2 heures.

D’autre part on a réparti les 4ml de sang total dans un tube de 10ml avec 4ml de PBS citraté. Le contenant a été agité par retournement à la main à une température comprise entre 20°C et 25°C pendant 4 minutes. Après avoir placé les tubes sur un portoir magnétique pendant 2 minutes, on a éliminé le surnageant et on a effectué des lavages cellulaires par l’addition de 1-2 ml de PBS citraté, à la fin on a ajouté 100µl de milieu nutritif. Pour compléter notre manipulation il était indispensable de mettre 1µl de la suspension cellulaire dans un puits de la plaque de Terasaki avec 1µl du complément et 5µl de Fluoro- Quench, afin de pouvoir vérifier la concentration cellulaire au microscope à fluorescence inversée ajusté à 2X 10^6^cellules/ml. Sous microscope la réaction positive montre des cellules mortes fluorescentes colorées en rouge (le bromure d’éthidium ne traverse pas la membrane des cellules intactes, il se lie à l’ADN des cellules mortes et émet la fluorescence rouge); en revanche La réaction négative montre des cellules vivantes colorées en vert (l’acridine orange traverse la membrane des cellules intactes, se lie à leur ADN et émet une fluorescence verte). Ces étapes ont été réalisées par l’étudiant chargé de l’étude.

**Étude statistique:** au cours de notre étude nous avons réalisé tous les typages HLA et nous avons sélectionné les plus pertinents. Les données ont été analysées en utilisant le programme SPSS version 17.0 en l’analyse bi variée qui consiste à la comparaison des pourcentages et cela par l’utilisation des tests de Chi 2 de Pearson et le test de Fisher. Dans le but de montré l’existence d’une éventuelle association entre les différents typages HLA et hypersensibilité allergique qui pourrait être un facteur déclencheur et/ou aggravant de l’asthme. Pour notre étude statistique on a utilisé les tests Qi deux et de Fisher exact avec un niveau de signification p<0.05; p<0.01 ou p<0.001 et avec K1 et K2 degré de liberté.

## Résultats

Le Tableau 1 comporte les caractères anthropométriques et physiologiques de notre population étudiée. Pour le Tableau 2 nos résultats montrent que le marqueur HLA DR11 (5) est significativement plus élevé chez les asthmatiques comparativement aux témoins avec une (p=0.05), vraisemblablement aux HLA-DR1 (p=0.31), HLA-DR3 (p = 0.92) et HLA-DR4 (p = 0.93) qui n’ont représenté aucune signification au même seuil (p-value < 0.05) (Tableau 2). De même, le marqueur HLA-DQ7 (3) (p=0.66) n’a représenté aucune différence significative aux seuils p < 0.05 ou p < 0,01, tandis que le marqueur HLA-DQ2 était très hautement significatif chez les asthmatiques que chez les témoins avec un p = 0.002. Quant au marqueur HLA-DQ6 (1) (p = 0.003), lui était nettement plus important chez la population témoin que chez les sujets asthmatiques avec une p < 0,01 (Tableau 3). Le Tableau 4 présente les résultats pour les deux marqueurs HLA-DR52, HLA-DR53 qui ne représentent aucune différence significative dont les valeurs étaient (p = 0.52), (p = 0.47) respectivement.

**Tableau 1:** Les caractères anthropométriques et physiologiques de la population étudiée

Paramètres		Asthmatiques (n=31)	Témoins (n=30)
Sexe	Féminin	20(64,50%)	6(20%)
	Masculin	11(35,50%)	24(80%)
Allergie	Oui	21(67,74%)	0(0,00%)
	Non	10(32,25%)	0(0,00%)
Age(ans)		43,29±17,83	40,36±18,50
Poids(Kg)		75,90±14,98	78,20±17,81
VEMS		73,23±9,49	86,01±8,13
VEMS/CVF (le rapport de Tiffeneau index)		76 ±10,35	87,05±6,45

VEMS: volume expiratoire maximal en une seconde, CVF: capacité vitale forcée, N: nombre de sujets, les valeurs sont représentées par moyenne ± écart-type

**Tableau 2:** Tableau récapitulatif du test du Chi 2 sur HLA- DR

HLA-DR	N Malades	Malade n (%)	N Témoins	Témoins n (%)	Chi2	P-Value
**DR 1**	31	5(16,12%)	30	8(26,66%)	1,000	0,31
**DR 3**	31	10(32,25%)	30	10(33,33%)	0,008	0,92
**DR 4**	31	8(25,80%)	30	8(26,66%)	0,005	0,93
**DR 11 (5)**	31	12(38,70%)	30	5(%16,66)	3,685	0,05*

HLA: Human Leukocyte Antigen, N et n: nombre de sujets,

* : différence significative p-value<0,05

**Tableau 3:** Tableau récapitulatif du test du Chi 2 sur HLA- DQ

HLA-DR	N Malades	Malade n(%)	N Témoins	Témoins n(%)	Chi2	P-Value
**DQ 2**	31	19(61,29%)	30	7(23,33%)	8,98	0,002**
**DQ 6 (1)**	31	7(22,58%)	30	18(60,00%)	8,82	0,003**
**DQ 7 (3)**	31	12(38,70%)	30	10(33,33%)	0,19	0,662

HLA: Human Leukocyte Antigenn N et n: Nombre de sujets,

** : différence très hautement significative p-value <0,01

**Tableau 4:** Tableau récapitulatif du test du Chi 2 sur HLA- DR52/53

HLA-DR	N° Malades	Malade(%)	N Témoins	Témoins n(%)	Chi2	P-Value
**DR 52**	31	13(41,93%)	30	15(50%)	0,39	0,52
**DR 53**	31	12(38,70%)	30	9(30%)	0,51	0,47

HLA: Human Leukocyte Antigen, N° et n°: Nombre de sujets, *: différence significative

## Discussion

Le principal objectif de ce travail était de déterminer un profil génétique ou un groupage tissulaire pour une population asthmatique constantinoise afin de permettre la confirmation ou l’infirmation de l’existence d’une association positive entre l’asthme et le système HLA de classe II. Comme le montre notre étude statistique, rejoignant ainsi les résultats obtenus par une étude italienne réalisée par C. Di Somma *et al.* en 2003 [8] à l’Institut de génétique et de biophysique à Naples en Italie, le marqueur HLA DR11 était significativement plus élevé chez les asthmatiques par rapport aux témoins avec une p-value < 0,05. De même, le marqueur HLA-DQ2 était très hautement significatif chez les asthmatiques que chez les personnes saines avec une p < 0,01, dans ce même contexte il convient de citer l’étude de GAO Jinming *et al.* en 2003 [9] au laboratoire national de biologie moléculaire à Pékin qui a apporté les même résultats que notre étude. Ceci nous conduit à penser qu’il s’agit peut-être de marqueurs HLA en association avec l’asthme, nos résultats sont controversés par l’étude menée par A. P. Knutsen *et al.* en 2010 qui suggère que le marqueur HLA-DQ2 a un effet protecteur [10].

La comparaison de nos résultats avec ceux obtenus grâce à d’autres études retrouvées en littérature nous ont conduit à dire que ces gènes pourraient être prédisposant à l’asthme, autrement dit, ils contribueraient à la pathogénèse de la maladie, on parle alors de gènes de susceptibilité. En revanche le marqueur HLA-DQ6 (1) était nettement plus important chez la population témoin que chez les sujets asthmatiques avec une p-value<0,01, il s’agit donc d’un marqueur qui pourrait avoir un effet protecteur contre l’apparition de la maladie, ce qui a également été retrouvé dans l’étude chinoise citée auparavant [11]. La régulation de la réponse immune par le locus HLA de classe II (locus D) reste obscure. Le locus HLA D est très polymorphe, notamment dans la partie qui correspond aux régions hypervariables des gènes A et B de chaque sous-région HLA D (DR, DP, DQ). Ces zones sont importantes pour la liaison entre l’allergène et les molécules HLA D portées par les cellules présentant l’antigène. Nous devons préciser que si la taille de notre échantillon était plus importante notre étude aurait été plus intéressante et peut être d’autres marqueurs HLA retrouvés en littérature comme le HLA-DR3 et le HLA-DR7 auraient été hautement significatifs chez les patients asthmatiques [12].

## Conclusion

Étant donné la nature complexe de l’asthme qui implique des facteurs génétiques et environnementaux dans son développement, une analyse d’association du système HLA de classe II avec l’asthme a été conduite sur une population constantinoise. Les résultats ont montré une association significative entre l’asthme et les marqueurs HLA-DR11 et HLA-DQ2 qui sont considérés comme des gènes de susceptibilité contribuant à la pathogénèse de la maladie.

### Etat des connaissances actuelles sur le sujet

- Le complexe majeur d’histocompatibilité est un des molécules indispensable dans l’interaction des cellules dendritiques et lymphocytes T lors de la présentation d’un antigène/allergène dont le 1er signal, qui confère la spécificité, est fourni par la présentation de l’antigène via les molécules HLA de type II;
- Un locus centré sur le marqueur rs9273349 situé dans la région HLA-DQ (6p21) a été associé à l’asthme débutant à l’âge adulte et des variants génétiques de la région HLA de classe II dans l’asthme de l’adulte, et leurs interactions avec les expositions professionnelles à des allergènes de haut poids moléculaire ont été étudiées dans étude épidémiologique des facteurs génétiques et environnementaux de l’asthme, l’hyperactivité bronchique et l’atopie (EGEA).

### Contribution de notre étude à la connaissance

- Permettra de mieux comprendre les mécanismes physiopathologiques de l’asthme avec l’implication de gènes codant des protéines décrites pour initier une réponse inflammatoire de profil Th2;
- Déterminer un profil génétique ou un groupage tissulaire pour une population asthmatique constantinoise;
- Permettra la confirmation ou l’infirmation de l’existence d’une association positive entre l’asthme et le système HLA de classe II.

## Conflits d’intérêts

Les auteurs ne déclarent aucun conflit d’intérêts.
